# Inhibition of HIV-1 replication with stable RNAi-mediated knockdown of autophagy factors

**DOI:** 10.1186/1743-422X-9-69

**Published:** 2012-03-16

**Authors:** Julia JM Eekels, Sophie Sagnier, Dirk Geerts, Rienk E Jeeninga, Martine Biard-Piechaczyk, Ben Berkhout

**Affiliations:** 1 Laboratory of Experimental Virology, Department of Medical Microbiology, Center for Infection and Immunity Amsterdam (CINIMA), Academic Medical Center of the University of Amsterdam, Meibergdreef 15, 1105, AZ Amsterdam, The Netherlands; 2 Centre d'études d'agents Pathogènes et Biotechnologies pour la Santé, CPBS UMR5236 CNRS UMSF, 1919 Route de Mende, 34293 Montpellier, France; 3 Department of Human Genetics, Academic Medical Center of the University of Amsterdam, Meibergdreef 15, 1105, AZ Amsterdam, The Netherlands; 4 Department of Pediatric Oncology/Hematology, Sophia Children's Hospital, Erasmus University Medical Center, Dr. Molewaterplein 60, 3015, GJ Rotterdam, The Netherlands

**Keywords:** HIV-1, Autophagy, RNAi, Antiviral

## Abstract

Autophagy is a cellular process leading to the degradation of cytoplasmic components such as organelles and intracellular pathogens. It has been shown that HIV-1 relies on several components of the autophagy pathway for its replication, but the virus also blocks late steps of autophagy to prevent its degradation. We generated stable knockdown T cell lines for 12 autophagy factors and analyzed the impact on HIV-1 replication. RNAi-mediated knockdown of 5 autophagy factors resulted in inhibition of HIV-1 replication. Autophagy analysis confirmed a specific defect in the autophagy pathway for 4 of these 5 factors. We also scored the impact on cell viability, but no gross effects were observed. Upon simultaneous knockdown of 2 autophagy factors (Atg16 and Atg5), an additive inhibitory effect was scored on HIV-1 replication. Stable knockdown of several autophagy factors inhibit HIV-1 replication without any apparent cytotoxicity. We therefore propose that targeting of the autophagy pathway can be a novel therapeutic approach against HIV-1

## Background

**Figure 1:**
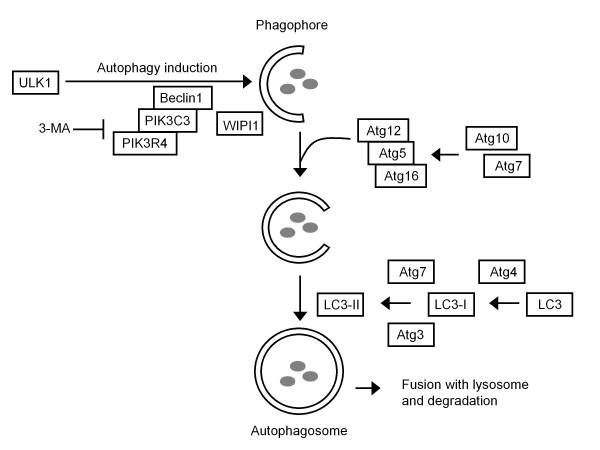
**Autophagy factors and their function in the autophagy pathway**. Autophagy can be induced by e.g. starvation signals. Two complexes are needed to form the phagophore. One includes ULK1, the other the class III phosphatidylinositol 3-kinase (PIK3C3), PIK3R4 and Beclin 1, together with WIPI1. To form the autophagosome, two conjugation systems play a role. The Atg12-Atg5 system forms a complex with non-covalently bound Atg16. The conjugation of LC3-I (LC3 cleaved by Atg4) with PE generates LC3-II. This process requires Atg7 and Atg3. The Atg12-Atg5-Atg16 complex is detected only during the membrane formation stage; LC3-II is detected at each step of autophagosome formation. The autophagy inhibitor 3-methyladenine (3-MA) acts on the class III phosphatidylinositol 3-kinase.

Autophagy is a cellular process leading to the degradation of cytoplasmic components, such as long-lived proteins and organelles [1]. The process starts with the engulfment of portions of the cytoplasm within a phagophore, eventually forming a double-membrane organelle called the autophagosome (Figure 1). The autophagosome subsequently fuses with lysosomes and the contents are degraded. Autophagy is mostly known as a cellular recycling mechanism in the event of nutrient starvation, but the process has also been implicated in i.e. developmental control, tissue homeostasis, tumor suppression and antigen-presentation [2-5]. Autophagy has several functions in immunity, as it not only eliminates cellular components, but intracellular pathogens like viruses as well. Not surprisingly, several viruses have evolved countermeasures to evade or neutralize this pathway [6,7]. For example, herpes simplex virus 1 (HSV-1) blocks two steps in the autophagy pathway with a single viral protein: ICP34.5, thereby preventing degradation of newly formed virus [8,9].

On the other hand, some viruses need autophagy to complete their replication cycle. Several positive-stranded RNA viruses such as poliovirus remodel intracellular membrane structures as scaffolds for their replication machinery [10]. These membranous structures are thought to be autophagic vacuoles. For influenza A virus, two studies highlight two different aspects of the complex interaction between the invading virus and autophagy. One study reported that the intracellular concentration of autophagy marker protein LC3-II increased during influenza virus infection and pharmacological inhibition of autophagy reduced the viral titers, indicating that influenza requires autophagy [11]. However, it has also been shown that influenza virus arrests autophagosome degradation, for which the viral M2 protein is solely responsible. This block of autophagy makes the infected cells more susceptible to apoptosis [12].

In case of human immunodeficiency virus type 1 (HIV-1), it is not clear to what extent autophagy influences the viral replication cycle, or whether the virus influences the autophagy pathway. It has been reported that the expression of HIV-1 Envelope protein (Env) on the surface of infected cells induces autophagy in bystander cells through gp41-mediated membrane fusion [13]. The induction of autophagy subsequently leads to death of these uninfected cells [13,14]. This mechanism has been used to explain the so-called "bystander-effect", which is the massive depletion of uninfected cells in HIV-1 infected individuals. Two studies also indicated that HIV-1 inhibits autophagy in the infected CD4^+ ^T cell, shown by reduced expression of the two autophagy marker proteins LC3 and Beclin1 and analysis of infected cells by electron microscopy [15,16]. Furthermore, the viral Nef protein prevents destruction of HIV-1 components in autolysosomes, thus blocking the antiviral role of autophagy in macrophages [17]. Several autophagy factors were identified in a transient genome-wide RNAi screen for cellular co-factors of HIV-1 replication [18], suggesting that HIV-1 also needs autophagy or at least some of its components to complete the replication cycle. Indeed, stable knockdown of one of the identified autophagic co-factors, Atg16, resulted in long-term inhibition of HIV-1 replication [19]. Also in primary cells it has been shown that several autophagy factors, e.g. Beclin1, Atg5 and Atg7 are required for HIV-1 replication [17,20]. If HIV-1 indeed requires autophagy for its replication, inhibiting the pathway could be of therapeutic use. Since the virus relatively easily gains resistance against drugs targeting viral components, it has been hypothesized that targeting of cellular co-factors would make it more difficult for the virus to gain resistance [21,22]. Therefore we sought to inhibit several autophagy factors (ATGs) via RNA interference (RNAi). Stable knockdown cell lines were generated, each cell line expressing a short hairpin RNA (shRNA) against a specific mRNA encoding an autophagy factor. Thirteen autophagy factors were tested, distributed along the autophagy pathway (Figure 1). Atg1/unc-51-like kinase (ULK1) is required for the initiation of autophagy. Autophagosome biogenesis is coordinated by the phosphatidylinositol 3-kinase (PIK3) complex containing the PIK3 p100 subunit (PIK3C3) and the PI3K p150 subunit (PIK3R4), which associate with Beclin1. WIPI1 regulates the transport of phosphatidylinositol-3-phosphate to the membranes. Two ubiquitin-like conjugation complexes are required; the first one forms a complex between Atg5 and Atg12, and Atg16 is non-covalently bound to this complex in a process that is catalyzed by Atg7 and Atg10. In the second conjugation system, LC3 is cleaved by Atg4 cystein proteases, essentially Atg4A and Atg4B, making it possible for Atg7 and Atg3 to generate the phosphatidylethanolamine (PE)-bound form of LC3: LC3-II.

We show that HIV-1 replication can be delayed in stable ATG knockdown cell lines. An additive inhibitory effect was observed when two ATGs were knocked down simultaneously, thus stressing the therapeutic potential of this strategy. Importantly, this HIV-1 replication delay was not accompanied by RNAi-induced cytotoxicity, suggesting that autophagy can be targeted in host cells without serious side effects.

## Results

### Stable knockdown of ATG proteins inhibits HIV-1 replication

**Figure 2:**
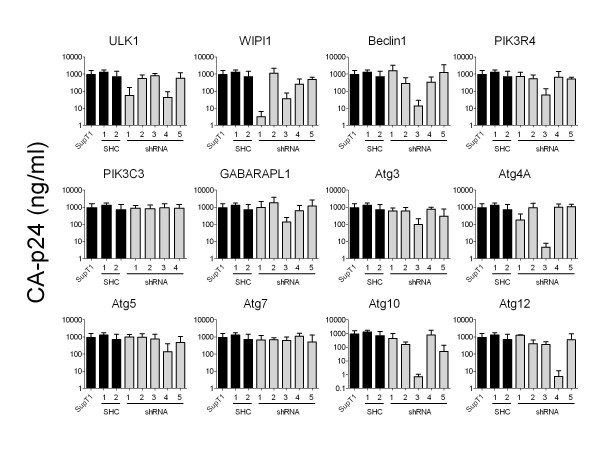
**Screen of shRNAs against 12 autophagy factors**. Stable knockdown cell lines with shRNAs against 12 ATGs were generated and challenged with HIV-1. Control cells are untransduced SupT1 cells, SHC1 cells (empty vector) and SHC2 cells, expressing a scrambled shRNA. The screen was repeated twice and each infection was performed in duplicate. The concentration of viral CA-p24 protein (ng/ml) in the culture supernatant at peak infection (day 10) was measured. CA-p24 concentrations were normalized with the Factor Correction program [39] and the average is plotted, error bars represent standard deviation from the mean.

To test whether stable knockdown of individual autophagy factors has an effect on HIV-1 replication, we generated cell lines expressing a single shRNA against a specific mRNA encoding one of the 12 autophagy factors (ATGs). Per autophagy factor 4 or 5 shRNAs were tested, resulting in 61 cell lines including 2 controls. The controls were the empty lentiviral vector SHC1 and the vector encoding a scrambled shRNA without a known mRNA target (SHC2). Testing of multiple shRNAs per ATG has several advantages. First, it allows one to score a similar phenotype for different shRNAs that target the same factor, which helps to determine whether the effect is specific. Second, as different shRNAs provide different knockdown efficiencies, the chance that at least one shRNA induces a sufficient knockdown of the specific target increases. To increase the chance of observing antiviral activity, we used a relatively high multiplicity of infection (MOI) of the lentiviral vectors. Because no shRNAs were available against LC3, we included shRNAs against GABARAPL1, a paralogue of LC3 that has been shown to function in autophagy [23]. All cell lines were tested for inhibition of HIV-1 replication in three independent experiments performed in duplicate. Stable cells were challenged with HIV-1 and the accumulation of CA-p24 in the culture supernatant was monitored. We measured the average CA-p24 concentration at peak infection, corresponding to 10 days post infection. In 14 cell lines at least a log decrease in CA-p24 levels and thus virus replication was observed. Inhibition was measured for the shRNAs ULK1-1 and 4, WIPI1-1 and 3, Beclin1-3, PIK3R4-3, GABARAPL1-3, Atg3-3, Atg4A-1 and 3, Atg5-4, Atg10-3 and 5 and Atg12-4 (Figure 2).

**Figure 3:**
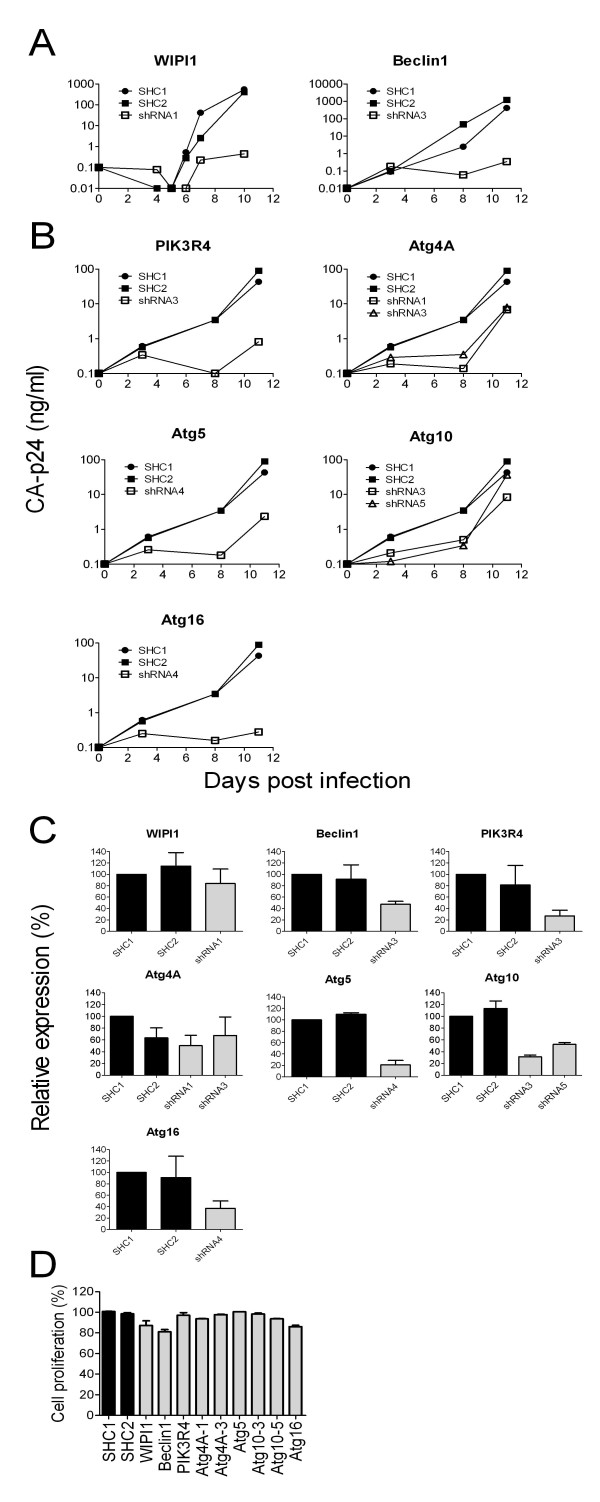
**Analysis of HIV-1 replication, ATG-mRNA levels and cell growth in stable knockdown cells**. **A**. Inhibition of HIV-1 replication as observed in Figure 2 was confirmed in newly generated knockdown cells for Beclin1 and WIPI1. CA-p24 concentration was followed for 10-11 days post infection. **B**. In a second experiment HIV-1 inhibition was confirmed for PIK3R4-1, Atg4A-1 and 3, Atg5-4, Atg10- 3 and 5 and Atg16-4. **C**. The mRNA knockdown was measured with RT-qPCR analysis for the indicated shRNAs. The level measured in the control SHC1 cells was set at 100%. **D**. Transduced cell mixtures were analyzed in the competitive cell growth assay, which is based on different growth rates of untransduced GFP^- ^versus transduced GFP^+ ^cells. Cell proliferation rates were compared to SHC1 cells, which was set at 100%.

To confirm these results, cells freshly transduced with the suppressive shRNA cassettes were again challenged with HIV-1. Virus replication was monitored over a period of 11 days by CA-p24 measurement in the culture supernatant. Inhibition was confirmed for the shRNAs WIPI1-1 and Beclin1-3 (Figure 3A) and in an independent experiment for PIK3R4-3, Atg4A-1 and 3, Atg5-4, Atg10-3 and 5 and Atg16-4 (Figure 3B), but HIV-1 inhibition could not be confirmed for other shRNAs (ULK1-1 and 4, WIPI1-3, GABARAPL1-3, Atg3-3 and Atg12-4). The Atg16 results do confirm earlier results on specific inhibition of HIV-1 replication [19]. For those cases where no inhibition of HIV-1 replication was observed, it remains unclear whether this is due to insufficient knockdown of the target ATG or that the ATG is dispensable for HIV-1 replication. Further experiments were conducted with the 9 shRNAs against 7 different ATGs that showed a reproducible inhibitory effect in multiple experiments performed with two independently transduced cell samples. In other words, all shRNAs that caused HIV-1 inhibition in one of the two experiments were abandoned.

For the selected shRNAs we first determined the ATG mRNA knockdown efficiency by RT-qPCR (Figure 3C). The results demonstrate a good knockdown (60-80%) for PIK3R4, Atg5, Atg10-3 and Atg16. A modest reduction in mRNA level (40-60%) was scored for Beclin1, Atg4A-1 and 3 and Atg10-5. For WIPI1 only a small reduction in mRNA expression (less than 20%) was measured. These results suggest that the knockdown level required for HIV-1 inhibition may vary per co-factor.

We determined the effect of ATG silencing on cellular autophagy by analyzing the level of LC3-II (LC3 bound to autophagic membranes) in the different cell lines. The level of LC3-II was analyzed under nutrient-rich and starvation conditions in the presence and absence of anti-proteases to analyze the autophagic flux. Starvation signals lead to conversion of LC3-I to LC3-II and the presence of protease inhibitors prevents lysosomal degradation such that LC3-II accumulates in the cell. Starvation-induced autophagy is functional in cell lines expressing a shRNA against WIPI, Beclin1 and Atg10. The results are summarized in Table 1. In contrast, the autophagy flux is impaired in cells expressing PI3KR4, Atg5 or Atg16 shRNAs. The autophagy process is dramatically altered when Atg4 expression is shut down.

**Table 1:** Autophagy activity upon ATG knockdown

Targeted ATG	Starvation-induced LC3 lipidation	Autophagy flux
WIPI1	_+_^a,b^	+

Beclin1	+	+

PIK3R4	+	- ^c^

Atg4A-1	+	+

Atg4A-3	+/- ^d^	-

Atg5	+	-

Atg10-3	+	+

Atg10-5	+	+

Atg16	+	-

^a ^Results are representative of two separate experiments

^b ^+; LC3-II increase similar to that in control SupT1 cells

^c ^-; no LC3-II increase

^d ^+/-; minor LC3-II increase

To score for effects on cell proliferation, we performed the competitive cell growth or CCG assay [24]. For that purpose the puromycin selection marker in the lentiviral constructs was replaced by the eGFP marker that allows the detection of transduced cells. Upon lentiviral-mediated transduction, the mixed culture of GFP^+ ^and untransduced GFP^- ^cells is followed longitudinally by FACS analysis. Based on the known doubling time of untransduced GFP^-^SupT1 cells, the decrease in GFP^+^/GFP^- ^ratio over time was used to calculate the relative cell growth capacity, with the proliferation of control SHC1 cells set at 100% (Figure 3D). Knockdown of Atg5 has no effect on cell proliferation. A small cell growth reduction of less than 10% was observed for PIK3R4, Atg4A (both shRNAs) and Atg10 (both shRNAs). Three shRNAs (Beclin1, WIPI1 and Atg16) induce more significant cytotoxicity with a 10-20% reduced cell growth rate. Beclin1 and WIPI1 were abandoned after this point because the effect on HIV-1 replication may be caused by the cytotoxic effect of these shRNAs.

### Knockdown of autophagy factors inhibits production of viral particles

**Figure 4:**
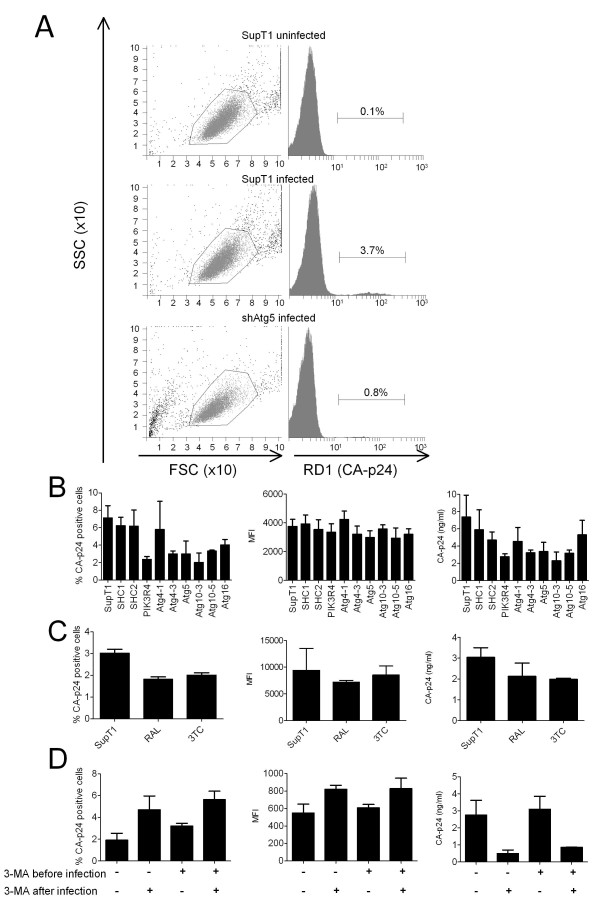
**Single cycle infection experiments**. **A**. ATG knockdown cells were infected with HIV-1 for 4 h, excess virus was washed away and new infections were prevented by addition of the fusion inhibitor T1249. The percentage of CA-p24 positive cells was measured at 48 post infection. The live cell population was determined by forward and side scatter (left panels). Cells were intracellularly stained for CA-p24 with a specific antibody labeled with RD1 (right panels). Mock infected SupT1, infected SupT1 and infected shAtg5 cells are shown as examples. **B**. FACS data for all tested cells. The percentage of CA-p24 positive cells was measured at 48 h post infection by FACS (left panel). The mean production of CA-p24 per positive cell is represented as the mean fluorescence intensity (middle panel). The concentration of CA-p24 in the culture supernatant was determined by ELISA (right panel). **C**. To test whether indeed early HIV-1 replication steps were inhibited, the Reverse Transcriptase 3TC drug and the Integrase inhibitor Raltegravir (RAL) were tested in single cycle infection experiments. **D**. SupT1 cells were either mock treated or incubated with 3-MA either starting 4 h before infection or for 48 h post infection, or both. Cells were analyzed for the percentage of CA-p24 positive cells (left panel), MFI (middle panel) and CA-p24 concentration in the culture supernatant (right panel). All single cycle infections were performed three times and per experiment every infection was performed in triplicate. A representative experiment is shown, bars represent the mean and error bars the standard deviation from the mean.

Single cycle infection experiments were performed to determine which stage of the HIV-1 replication cycle is blocked in the ATG knockdown cells. Knockdown and control cells were incubated with HIV-1 for 4 h, the virus was washed away and new infections were prevented by addition of the fusion inhibitor T1249 to the culture medium. The percentage of cells positive for intracellular CA-p24 and the concentration of CA-p24 in the culture supernatant were measured at 48 h post infection by FACS (Figure 4A). This allows the establishment of integrated provirus that can express new viral proteins. For a single shRNA (Atg4A-1) no significant difference was measured. For 6 shRNAs a reduction in both the intracellular and extracellular CA-p24 level was measured: PIK3R4, Atg4A-3, Atg5, Atg10-3, Atg10-5 and Atg16 (Figure 4B, left and right panel). A reduction in the number of CA-p24 positive cells means that less cells were productively infected by HIV-1, which should also result in reduced CA-p24 levels in the culture supernatant. The mean production of CA-p24 per positive cell, shown by the mean fluorescence intensity (MFI), was not affected (Figure 4B, middle panel). These combined results indicate an early block (from virus entry to transcription) in the HIV-1 replication cycle in these shRNA-expressing cells. To test whether we indeed probe for early replication steps, the Reverse Transcriptase drug 3TC and the Integrase inhibitor Raltegravir were tested in single cycle infection experiments (Figure 4C). This produced a pattern similar to that of the shAtg cells, that is a reduction in the percentage of CA-p24 positive cells and CA-p24 concentration in the culture supernatant, but no effect on CA-p24 production per infected cell as measured by the MFI. We therefore conclude that knockdown of ATG factors blocks an early HIV-1 replication step.

As an alternative means to inhibit the autophagy pathway, the autophagy inhibitor 3-methyladenine (3-MA) was used, which blocks the activity of the PIK3C3 and PIK3R4 kinases (Figure 1). First, 3-MA was tested in single cycle infection experiments on wild type SupT1 cells. Cells were pre-treated with 3-MA for 4 h, after which the drug was washed away before HIV-1 infection. Alternatively, cells were first infected with HIV-1 and then treated for 48 h. We also tested a combination of these treatments. All treated cell cultures were compared to the untreated control cells (Figure 4D). As reported earlier, we observed increased cell death in cultures that were treated with 3-MA for a prolonged period, including the samples that received 3-MA for 48 h post infection [17]. Treating cells before infection did not cause significant changes in cell viability. The concentration of CA-p24 in the culture supernatant was dramatically reduced when cells were treated with 3-MA after infection (Figure 4D, right panel). However, more cells in the culture became CA-p24 positive and the mean production of CA-p24 per actively infected cell was slightly increased (Figure 4D, left and middle panel). Thus, cells treated with 3-MA post infection accumulate CA-p24 and yield a reduced CA-p24 concentration in the culture supernatant. These results indicate that a late replication step (from transcription to budding) is affected by 3-MA treatment after infection.

### Simultaneous knockdown of two ATGs enhances HIV-1 inhibition

To test whether the simultaneous knockdown of two ATGs is tolerated by cells and whether the HIV-1 inhibition can be boosted, we generated double-knockdown cells expressing shRNAs against Atg16 and Atg5. Atg16 was chosen as its knockdown resulted in strong inhibition of HIV-1 replication with limited cytotoxicity [19]. Atg5 knockdown resulted in good inhibition of HIV-1 replication without inducing cytotoxicity. Controls were untransduced SupT1 cells and the single-knockdown cell lines. The single knockdown cell lines were actually also transduced twice; the second transduction was performed with the SHC2 scrambled shRNA control. The first transduction used lentiviral vectors with the puromycin selection marker, whereas the second transduction was performed with vectors carrying a GFP-selection marker. To increase the change of scoring an additive effect, we purposely transduced cells at a relatively low multiplicity of infection (MOI) of 0.2. This will yield maximally 1 copy of each shRNA gene construct per doubly transduced cell, thus avoiding saturation of the RNAi mechanism.

**Figure 5:**
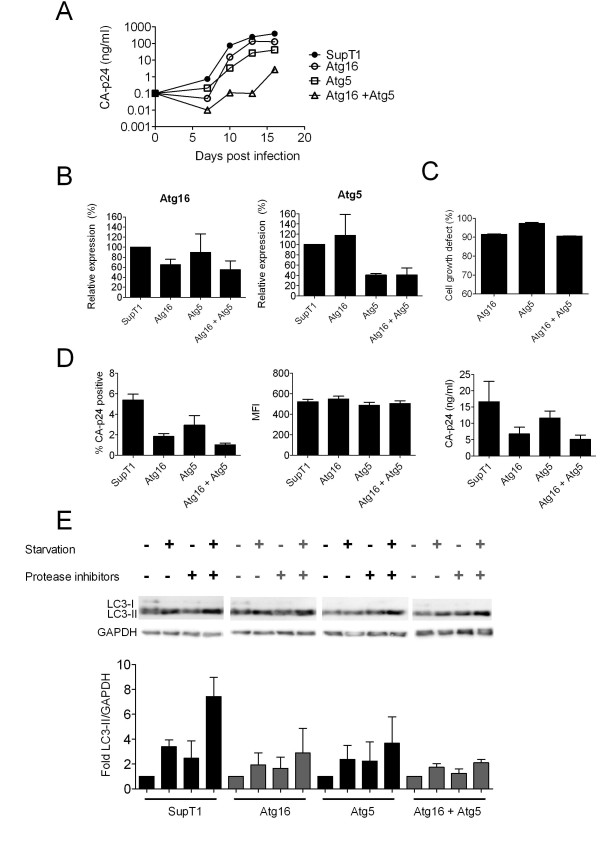
**Additive HIV-1 inhibition by simultaneous knockdown of Atg5 and Atg16**. **A**. Single and double knockdown cell lines were challenged with HIV-1 and replication was followed over time by measuring CA-p24 in the culture supernatant. B. Relative mRNA expression levels of Atg5 and Atg16 was determined by RT-qPCR. **C**. The effect of RNAi-mediated knockdown on cell growth was determined by the competitive cell growth assay. The percentage cell growth reduction is plotted. **D**. Single and double knockdown cells were used in single cycle infections. Cells were analyzed 48 h post infection for the percentage of CA-p24 cells (left panel), MFI (middle panel) and CA-p24 concentration in the culture supernatant (right panel). **E**. Autophagy function in the single and double knockdown cells was analyzed in nutrient-rich and starvation conditions (first 2 bars for each cell line) and for autophagic flux, where the degradation step of autophagy is inhibited by protease inhibitors (last 2 bars for each cell line). The intensity of the LC3-II signal was corrected for the GAPDH level on Western blot.

Cell lines were challenged with virus and a clear delay of HIV-1 replication was observed in the double-knockdown cell line (Figure 5A). In this low MOI setting, HIV-1 replication was not greatly delayed in the single knockdown cells. Knockdown of Atg5 and Atg16 was measured by RT-qPCR (Figure 5B) and knockdown of both Atg5 and Atg16 was confirmed in the double-knockdown cells. All transduced cells express GFP and could thus be tested in the competitive cell growth assay. The cell growth defect of the double-knockdown cell line is comparable to that observed when Atg16 knockdown is combined with the scrambled shRNA (Figure 5C). Thus, cell proliferation does not seem hampered more severely by knockdown of two ATGs. In the single cycle replication assay the additive effect of double knockdown of Atg5 and Atg16 was again observed. Fewer cells became positive for CA-p24 and the concentration of CA-p24 in the culture supernatant was concomitantly reduced when compared to the single-knockdown cells and SupT1 cells (Figure 5D).

Cells were incubated for 2 h in minimal medium to generate starvation signals in the absence and presence of protease inhibitors. The LC3-II level was detected by Western blot and normalized against the housekeeping protein GAPDH. Starvation-induced autophagy and autophagic flux were reduced in the single-knockdown cells and dramatically reduced in the double-knockdown cells (Figure 5E).

## Discussion

In this study we show that stable RNAi-mediated knockdown of autophagy factors leads to inhibition of HIV-1 replication. Multiple shRNAs against 13 autophagy factors (ATGs) were tested and inhibition of virus replication was scored for 7 ATGs. We confirmed knockdown of the targeted mRNAs and performed additional cell proliferation and autophagy tests, which allowed us to conclude that RNAi against PIK3R4, Atg4A, Atg5 and Atg16 results in HIV-1 inhibition due to a block of autophagy. For these 4 ATGs a specific reduction in the target mRNA expression level and a clear defect in the autophagy pathway were measured. For Atg10-1 and 3 no block in autophagy was observed, but a specific reduction in mRNA expression levels was measured and a minor effect on cell proliferation was observed. It is possible that the effect on HIV-1 replication is due to a cellular function of Atg10 other than its role in autophagy. Cell proliferation was not altered upon Atg5 knockdown, and only a small effect on cell growth was observed for PIK3R4 and Atg4A. Cell proliferation of Atg16 knockdown cells was 15% reduced compared to untransduced cells, but for this shRNA a specific reduction in Atg16 mRNA expression levels and a clear defect in autophagy were observed. For Beclin1 and WIPI1 a delay of HIV-1 replication was measured despite the absence of an effect on autophagy, and these shRNAs were therefore not selected for follow-up studies. The effect on HIV-1 replication could be due to the adverse effects on cell proliferation. An alternative explanation is that these specific shRNAs induce an Interferon alpha (IFNα) response that inhibits HIV-1 replication [25]. An earlier genome-wide knockdown study has already identified ATG factors necessary for HIV-1 replication [18], although this was not confirmed in a second RNAi screen [26]. However, none of the ATGs for which we scored inhibition of HIV-1 replication were identified in the knockdown study by Brass et al., except for Atg16. In primary cells it has been shown that knockdown of Beclin1 and Atg5 [20] or Beclin1 and Atg7 [17] results in inhibition of HIV-1 replication.

When expression of Atg5 and Atg16 was inhibited simultaneously, additive HIV-1 inhibition was measured. Knockdown of both ATGs did not have a greater impact on cell proliferation than that observed in the singly transduced cells. Atg16 knockdown seems to be the sole determinant of cell growth delay in the double knockdown cells, which may be surprising as both Atg5 and Atg16 are involved in the same step of the autophagy pathway (Figure 1). Such an additive effect was measured for autophagy activity, which indicates that the modest delay in cell growth measured for Atg16 knockdown is not directly related to the impact on the autophagy pathway.

When ATG knockdown cells were analyzed in single cycle infection experiments, we observed that less cells did produce intracellular CA-p24 and the concentration of CA-p24 in the culture supernatant was concomitantly reduced. This indicates that less cells became productively infected. The mean CA-p24 production per CA-p24 positive cells (mean fluorescence intensity or MFI) was not affected by ATG knockdown. Thus, fewer cells are productively infected, but these cells do synthesize as much CA-p24 as control cells. This result indicates that ATG knockdown leads to an early block of HIV-1 replication (e.g. entry or reverse transcription).

As an alternative to blocking autophagy with RNAi, we tested the inhibitor 3-methyladenine (3-MA). Surprisingly, we observed markedly different results in single cycle infection experiments with 3-MA compared to the shRNA-expressing cell lines. First of all, we observed that a higher percentage of the cells became positive for intracellular CA-p24, whereas a reduction was measured in the shRNA-expressing cell lines. As normally a certain percentage of HIV-1 infections become latent, this result could indicate that treatment with 3-MA results in less latently infected cells and more productively infected cells. A second observation was that cells treated with 3-MA after HIV-1 infection exhibited an increased MFI, meaning that cells produced more CA-p24 per cell than untreated cells. Less CA-p24 was measured in the culture supernatant. This is similar to what was described by Kyei et al., confirming their conclusion that 3-MA mediated blocking of autophagy leads to inhibition of virus budding into the culture supernatant. 3-MA has been used for several years as a specific inhibitor of autophagy, however, there is accumulating evidence that 3-MA can have pleiotropic effects, and the impact on autophagy should ideally be confirmed by alternative means such as shRNAs [27,28].

Autophagy is a cellular pathway that is important in many viral infections, thus blocking the autophagy pathway could be of therapeutic value. In addition to viral infections, blocking autophagy has been proposed as a new therapeutic approach against cancer. Cancerous cells appear to exhibit increased autophagy activity that provides a survival mechanism when the cell is treated with chemotherapy [29]. Blocking autophagy with 3-MA in combination with anti-cancer drugs has been used against several types of cancer, e.g. breast and colorectal cancer, and siRNAs to silence the ATGs Beclin1 and Atg5 have been tested against cervical cancer [30-32]. Therefore RNAi-mediated knockdown of autophagy factors could be of therapeutic value against viruses and other diseases. In this study, we used lentiviral vector-mediated delivery of shRNAs and this delivery system provides an attractive possibility to develop a durable therapy for HIV-1 patients. The ex vivo transduction of hematopoietic stem cells with lentiviral vectors expressing anti-viral and/or anti-co-factor shRNAs should guarantee the life-long generation of HIV-1 resistant cells, e.g. T cells and monocytes. This approach is the focus of further studies in our laboratory.

## Methods

### DNA constructs

pLKO.1 DNA constructs expressing a specific shRNA were from the MISSION™ TRC-Hs 1.0 library [33]. Constructs including the negative control constructs SHC001 and SHC002 (hereafter named SHC1 and SHC2) were obtained from Sigma-Aldrich as bacterial clones. Plasmid DNA was extracted using the Nucleobond Midiprep columns according to the manufacturer's instructions (Macherey-Nagel). Target sequences can be found on the website of Sigma-Aldrich [http://www.sigmaaldrich.com/life-science/functional-genomics-and-rnai/shrna/individual-genes.html]. The pLKO.1 constructs from the MISSION™ TRC-Hs 1.0 library contain a puromycin selection marker, which was replaced with the gene for enhanced eGFP (eGFP) as described earlier [24]

### Chemicals

The T1249 peptide (WQEWEQKITALLEQAQIQQEKNEYELQKLDKWASLWEWF, Pepscan Therapeutics) was obtained as 10 000 × stock solution [34]. The autophagy inhibitor 3-MA (Sigma-Aldrich) was diluted in 70% methanol and used at a final concentration of 10 mM. Protease inhibitors pepstatin A and E64d and the anti-LC3 antibody were purchased from Sigma-Aldrich. Raltegravir (MK-0518) was obtained from Bio-Connect services [35] and used at a final concentration of 1 nM. Lamivudine (3TC) was obtained from GlaxoWellcome and used at a final concentration of 33 pM.

### Cell lines

The human embryonic kidney cell line HEK293T was grown in DMEM, supplemented with 10% FCS, 100 U/ml penicillin and 100 μg/ml streptomycin. The human T cell line SupT1 was cultured in Roswell Park Memorial Institute (RPMI) medium, supplemented with 10% FCS, 100 U/ml penicillin and 100 μg/ml streptomycin.

### Lentiviral vector production and generation of stable knockdown cell lines

Lentiviral vectors were produced as described earlier [36]. In short, HEK293T cells were co-transfected with pLKO.1-shRNA and the packaging plasmids (pVSV-G, pMDL and pRev-RRE) with Lipofectamin 2000 (Invitrogen). The medium was refreshed 1 day after transfection and the culture supernatant was harvested the next day. Aliquots of the culture supernatant with the lentiviral vectors were stored at -80°C. A sample was tested in CA-p24 ELISA.

SupT1 cells were seeded in 24-wells format at 1 × 10^5 ^cells/well and transduced with a fixed amount of lentiviral vector. Excess virus was washed away 1 day after transduction and selection of stably transduced cells was started by adding puromycin to the medium at a final concentration of 1 μg/ml. In the case of GFP-expressing cell lines, transduced cells were FACS sorted.

### CA-p24 ELISA

Culture supernatant was heat-inactivated at 56°C for 30 min in the presence of 0.05% Empigen-BB (Calbiochem). The CA-p24 concentration was determined by a twin-site ELISA with D7320 (Biochrom) as the capture antibody and the alkaline phosphatase-conjugated anti-CA-p24 monoclonal antibody EH12-AP (International Enzymes) as the detection antibody. Detection was performed with the Lumiphos plus system (Lumigen) in a LUMIstar Galaxy luminescence reader (BMG Labtechnologies). Recombinant CA-p24 produced in a baculovirus system was used as reference standard.

### HIV-1 replication and single cycle infection

The HIV-1 molecular clone HIV-1_LAI _[37] was used to produce virus by transfection of HEK293T cells. HIV-1 production was measured by CA-p24 analysis in the culture supernatant. For HIV-1 replication studies, SupT1 cells were seeded in a 6-wells plate at 4 × 10^5 ^cells/well and infected with HIV-1 (0.2 ng CA-p24). HIV-1 replication was monitored by scoring for syncytia formation and longitudinal measurement of CA-p24 production in the culture supernatant.

For single cycle infection experiments SupT1 cells were incubated with HIV-1 for 4 hours. Excess virus was washed away and the cells were cultured in the presence of entry inhibitor T1249 (Pepscan) to block subsequent rounds of viral entry. Intracellular CA-p24 was analyzed by FACS and extracellular CA-p24 was measured by ELISA at 48 h post infection.

### Competitive cell growth assay and flow cytometry

To assess the cytotoxicity induced by knockdown of autophagy factors, we used the competitive cell growth or CCG assay as described earlier [24]. In brief, SupT1 cells were transduced with 0.1 or 1 μl lentiviral vector that expresses a shRNA and the GFP selection marker. The percentage of cells expressing GFP in the transduction mixture was analyzed longitudinally by FACS analysis. Twice weekly a sample of the culture was taken, cells were collected by centrifugation (4 min at 4,000 rpm, Eppendorf centrifuge) and resuspended in FACS solution (Phosphate buffered saline (PBS) + 2% FCS) and analyzed on FACScanto (BD Biosciences). The live cell population was determined with forward and side scatter. Fluorescence was normalized using unstained SupT1 cells. Based on the known doubling time of untransduced cells, the change in GFP^+^/GFP^- ^ratio over the course of the experiment can be used to calculate the cell growth defect (%) of the GFP^+ ^transduced cells [24].

For intracellular CA-p24 staining, cells were collected by centrifugation (4 min at 4,000 rpm, Eppendorf centrifuge) and fixed in 250 μl 4% formaldehyde for 5 min at room temperature. Cells were permeabilized with 500 μl BD Perm/Wash™ buffer (BD Pharmingen) and stained for at least 1 h at 4°C in 50 μl BD Perm/Wash™ buffer containing 5 μl 1:100 diluted antibody against CA-p24 conjugated with PE (monoclonal mouse, clone KC57, Coulter). Excess antibody was washed away by washing twice with 500 μl FACS solution, cells were resuspended in 250 μl FACS solution and analyzed on a FACScanto. Uninfected and unstained samples were used as negative controls.

### RT-qPCR

mRNA knockdown levels for specific ATG targets were analyzed by RT-qPCR. RNA was isolated from 0.5 × 10^6 ^cells with the RNeasy kit (Qiagen) according to the manufacturer's protocol, including the DNase I treatment on the column. 1 μg RNA was used for reverse transcription (Thermoscript, Invitrogen) with Oligo-dT primers and cDNA synthesis was performed at 50°C. The cDNA was diluted 100 times and 5 μl of the diluted sample was used as template in a SYBR Green based RT-qPCR (SYBR Green FAST PCR, Qiagen) with an ABI Prism 7,000 detection system (Applied Biosciences). Primers for target genes and the internal control β-actin were from the Quantitect primer assays (Qiagen). The ΔΔ*Ct *method was used to calculate relative mRNA expression as described earlier [38].

### Analysis of autophagy

Autophagy was induced by nutrient starvation (EBSS) for 2 h in presence or absence of the lysosome protease inhibitors E64d and pepstatin A (10 μg/ml each) to analyze the autophagy flux. To monitor the induction of autophagy, the relative amount of the PE-conjugated form (LC3-II) was determined by immunoblot analysis of whole-cell lysate using a rabbit polyclonal antibody against LC3. Cells were washed twice in PBS and lysed in buffer containing 50 mM Tris-HCl (pH 8), 1% Triton X-100, 100 mM NaCl, 1 mM MgCl2, 150 mM PMSF, and complete mini protease inhibitor cocktail (Roche Diagnostics). Cell lysates were electrophoresed in 12% SDS-PAGE and blotted to PVDF membranes. After a blocking step with PBS and 0.5% casein for 1 h at room temperature, blots were incubated overnight at 4°C with the anti-LC3 antibody in the blocking buffer. After 3 washes with PBS and 0.05% Tween, the blots were incubated for 1 h at room temperature with peroxidase-coupled antiserum diluted in blocking buffer. After further washes, the immune complexes were revealed by ECL (Millipore). The image capture was taken by the G:BOX camera system (Syngene) and intensity of the signals was analyzed with GeneTools software. The LC3-II signal was compared to that of the control housekeeping protein GAPDH.

## Competing interests

The authors declare that they have no competing interests.

## Authors' contributions

JE carried out the production of stable knockdown cell lines, virus infection experiments and competitive cell growth assays and wrote the manuscript together with MB and BB. SS performed the autophagy analyses and designed Figure 1. DG provided the shRNA-constructs and together with RE helped to draft the manuscript. All authors read and approved the final manuscript.
